# Comparisons of quantitative approaches for assessing microglial morphology reveal inconsistencies, ecological fallacy, and a need for standardization

**DOI:** 10.1038/s41598-022-23091-2

**Published:** 2022-10-28

**Authors:** Tabitha R. F. Green, Sean M. Murphy, Rachel K. Rowe

**Affiliations:** 1 Department of Child Health, University of Arizona College of Medicine-Phoenix, Phoenix, AZ USA; 2 Department of Integrative Physiology, University of Colorado, 2860 Wilderness Place, Boulder, CO 80301 USA

**Keywords:** Inflammation, Microglia, Neurology, Neurological disorders, Brain injuries

## Abstract

Microglial morphology is used to measure neuroinflammation and pathology. For reliable inference, it is critical that microglial morphology is accurately quantified and that results can be easily interpreted and compared across studies and laboratories. The process through which microglial morphology is quantified is a key methodological choice and little is known about how this choice may bias conclusions. We applied five of the most commonly used ImageJ-based methods for quantifying the microglial morphological response to a stimulus to identical photomicrographs and individual microglial cells isolated from these photomicrographs, which allowed for direct comparisons of results generated using these approaches. We found a lack of comparability across methods that analyzed full photomicrographs, with significant discrepancies in results among the five methods. Quantitative methods to analyze microglial morphology should be selected based on several criteria, and combinations of these methods may give the most biologically accurate representation of microglial morphology.

## Introduction

Microglia are immune cells exclusive to the central nervous system^1^. Actively, microglia sample the microenvironment and respond to injury or infection^2,3^. Microglia have a distinctive morphology that changes based on their reactivity and function^4^. Under non-inflammatory conditions, microglia have a highly ramified morphology with many sinuous branches stemming from a small cell body. After detection of a pathological stimulus, microglia rapidly change their morphology by shortening their processes and enlarging their cell body^5–7^. This morphological change occurs on a continuum and the extent of de-ramification is associated with the severity of the pathological stimulus^5^.

Morphological changes in microglia are associated with pathology severity; thus, immunohistochemical analyses that investigate microglial morphology are powerful methods for assessing the level of inflammation caused by an insult. Techniques frequently used to quantitatively assess changes in microglial morphology either obtain averaged measurements across photomicrographs or examine individual isolated microglial cells. However, it remains unclear if these differing approaches produce comparable results and, consequently, whether associated inferences are reliable and/or accurate. Indeed, a lack of standardization of data-generating, data collection, and analytical methods is among the primary contributors to the reproducibility crisis and translational failures in neuroscience^8,9^. Preclinical neuroscience research using animal models is particularly susceptible to reproducibility issues^10,11^. Although environmental variability across laboratories cannot be fully controlled, other factors, such as inadequate training in experimental design, inappropriate application of statistical analysis approaches, and untested ability of many of the data-generating and sampling methods used to address the same outcome measures, contribute to the irreproducibility of findings in translational studies^10^.

**Figure 1:**
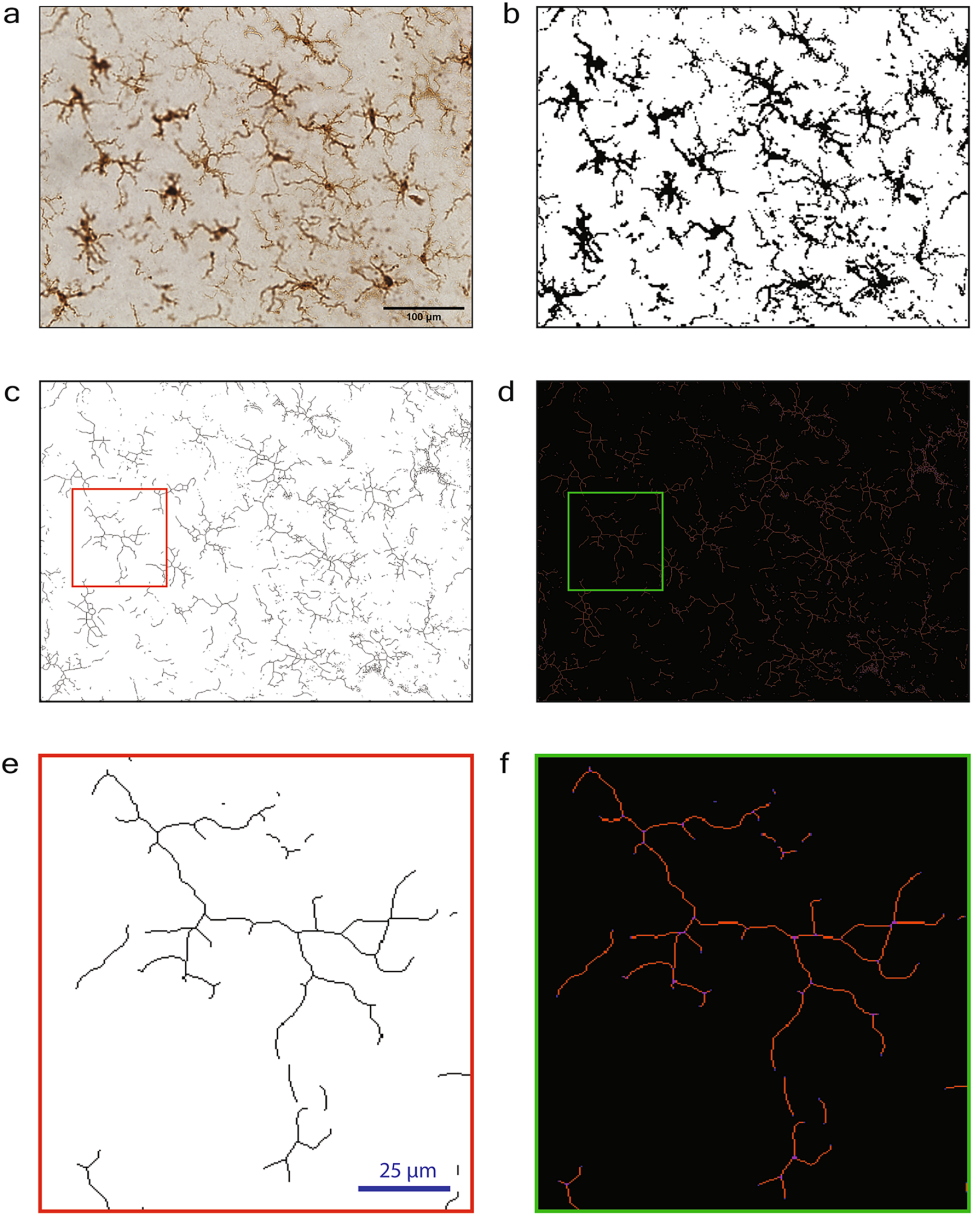
Full photomicrograph analysis techniques to assess microglial morphology in ImageJ (percent coverage and full photomicrograph skeletal analysis). (**a**) 40× photomicrograph of Iba1-stained microglia. (**b**) Iba1 photomicrograph converted to binary. (**c**) Iba1-binarized photomicrograph converted to a skeleton. (**d**) Tagged skeleton after the ‘analyze skeleton’ plugin. (**e**, **f**) Enlarged version of a skeletonized cell and a tagged skeleton from images (**c** and **d**), respectively. Scale bar = 100 µm.

**Figure 2:**
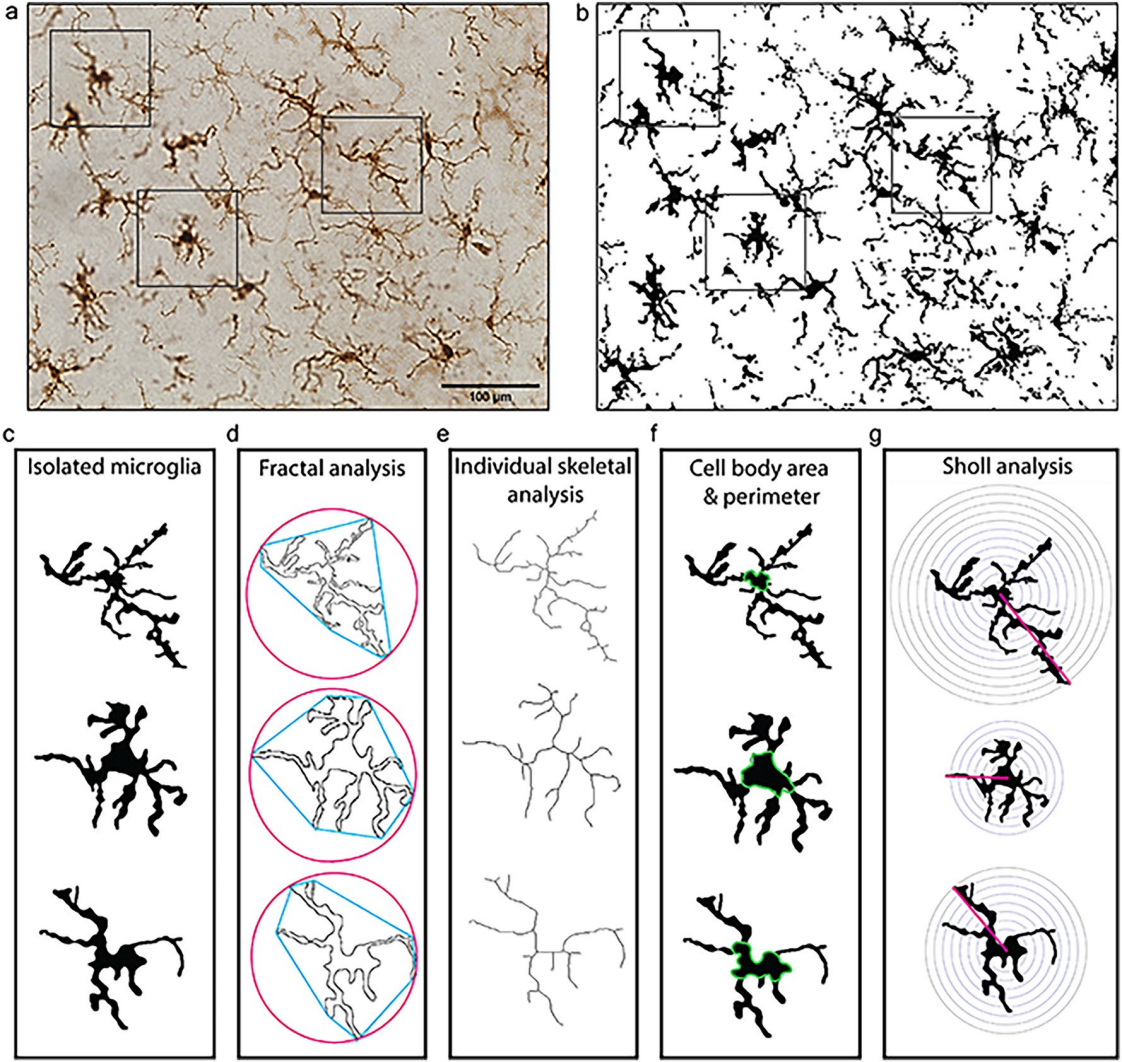
Single cell methods to assess microglial morphology in ImageJ. (**a**) Photomicrograph of Iba1-stained microglia. (**b**) Photomicrograph of Iba1-stained microglia in binary. Boxes indicate microglia that were randomly selected for isolation. (**c**) Binary isolated microglia. (**d**) For fractal analysis, isolated microglia were converted to outlines and analyzed using the FracLac plugin. (**e**) For individual skeletal analysis, microglia were skeletonized and analyzed using the skeletal analysis plugin. (**f**) Cell body area and perimeter were measured using the multipoint area selection tool. (**g**) Sholl analysis was performed to measure cell branching using concentric circles. Scale bar = 100 µm, applied in (**a**, **b**).

Herein, we applied five of the most commonly used ImageJ-based methods for quantifying the microglial morphological response to a stimulus to identical photomicrographs and isolated microglial cells from these photomicrographs, which allowed for direct comparisons among methods. Ionized calcium binding adapter molecule 1 (Iba1) is a microglial marker that is commonly used to examine microglial reactivity through morphological changes. We used two ImageJ-based full photomicrograph analysis techniques; percent coverage of Iba1 staining to show how much Iba1 positive staining is present in a given photomicrograph, and full photomicrograph skeletal analysis which calculates an averaged number of branches, branch endpoints, and branch length among cells in the field of view captured in a 40× photomicrograph^6,12–14^ (Fig. 1). Additionally, we used three single cell microglia analysis techniques that involved isolating individual, randomly selected microglial cells from a photomicrograph in ImageJ, and quantifying microglial morphology at a single cell level (Fig. 2). Fractal analysis was used to quantify the spatial complexity of the individually isolated microglia. Single cell skeletal analysis was used to quantify microglial ramification and cell body size. Sholl analysis, which uses intercepts on concentric circles around the cell body, was applied to determine the extent of branching an individual microglia has^15^. All five methods were applied to identify alterations in microglial morphology or percent coverage between a control group and a treatment group that had pharmacologically manipulated microglia.

## Results

Lipopolysaccharide (LPS) was given in this study as an inflammatory trigger to induce robust microglial reactivity, see “Methods” for experimental design. In the treatment group, microglia were depleted prior to LPS exposure and then repopulated before tissue collection. In the control group, microglia remained intact. Microglial reactivity was assessed using full photomicrograph skeletal analysis (Fig. 1) and at the single cell level using fractal analysis, individual skeletal analysis, and Sholl analysis (Fig. 2).

### Averaging among photomicrographs masked a significant difference in percent coverage of Iba1 between groups, whereas retaining individual photomicrographs detected the difference

**Figure 3:**
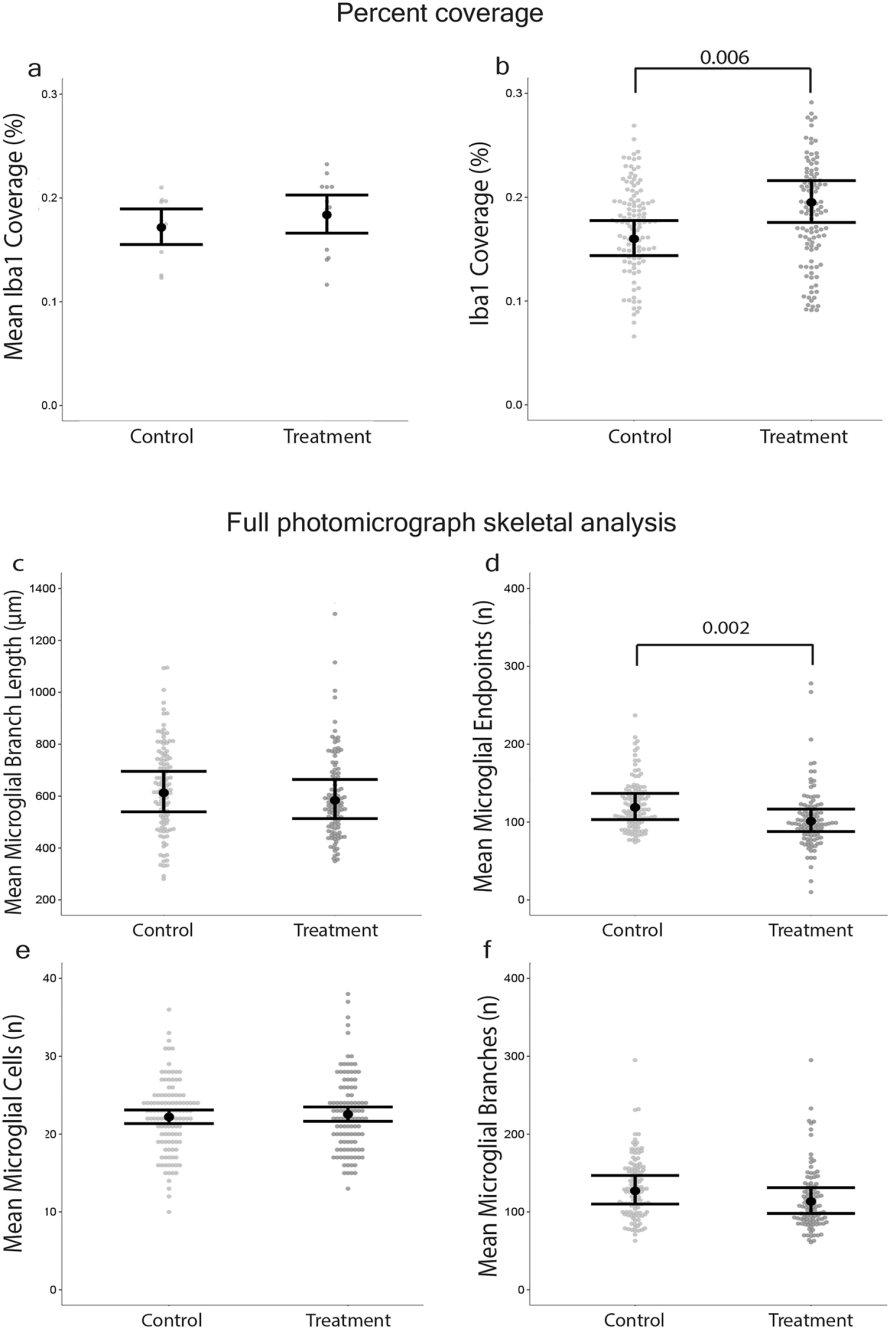
Full photomicrograph analyses of microglial morphology were not sensitive to differences between control and treatments groups. Photomicrograph-averaged Iba1 percent coverage masked a difference between control and treatment groups that photomicrograph-specific percent coverage detected. 3 brain slices per mouse and 3 photomicrographs per brain slice were used. (**a**) Mean percent coverage of Iba1 staining per animal. Treatment *n* = 12 mice, Control *n* = 13 mice. (**b**) Percent coverage of Iba1 staining, where individual data points (gray dots) were not averaged. Full photomicrograph skeletal analysis detected that microglia from the treatment group had fewer endpoints than the controls but did not detect differences in branch number or branch length. (**c**) Mean microglial branch length; (**d**) mean number of endpoints per cell per photomicrograph; (**e**) mean number of microglial cells per photomicrograph; and (**f**) mean number of microglial branches per cell per image. Individual data points are presented as gray dots. (**d**–**f**) Treatment *n* = 108 images (12 mice, 9 images/mouse), Control *n* = 117 images (13 mice, 9 images/mouse). Results are presented as point estimates with 95% confidence intervals, which were estimated from mixed effects models with a negative-binomial error distribution.

There was no difference between groups when the percent coverage of Iba1 per photomicrograph was calculated and the data from the 9 photomicrographs per mouse were averaged (*p*-value = 0.35, Mean ∆ = 1.2%, *d* = 0.0006; Fig. 3a). In contrast, significantly more Iba1 staining existed in the Iba1 images from the treatment group (microglia that had repopulated after depletion using PLX5622) compared to controls (intact microglia with no PLX5622 treatment) when the individual photomicrographs were retained as separate datapoints per mouse (*p*-value = 0.006, Mean ∆ = 3.5%, *d* = 0.002; Fig. 3b).

### Full photomicrograph skeletal analysis only detected that microglia in the treatment group had fewer endpoints than controls

Mean microglial branch length was calculated by averaging the total length of branches in the photomicrograph by the total number of microglia in the photomicrograph. Full photomicrograph skeletal analysis did not detect differences in mean microglial branch length between photomicrographs from the treatment groups (*p*-value = 0.40, Mean ∆ = 28 µm, *d* = 0.002 Fig. 3c). Photomicrographs from the treatment group indicated that microglia had fewer mean endpoints than control microglia (*p*-value = 0.002, Mean ∆ = 18, *d* = 0.01; Fig. 3d). A difference in mean number of microglia per photomicrograph between the treatment groups was not detected (*p*-value = 0.60, Mean ∆ = 0, *d* = 0.01; Fig. 3e). A difference in mean microglial branches per cell per photomicrograph between treatment groups was not detected (*p*-value = 0.08, Mean ∆ = 13, *d* = 0.005; Fig. 3f).

### Fractal analysis detected that microglia from the treatment group had a less complex branching pattern than controls

**Figure 4:**
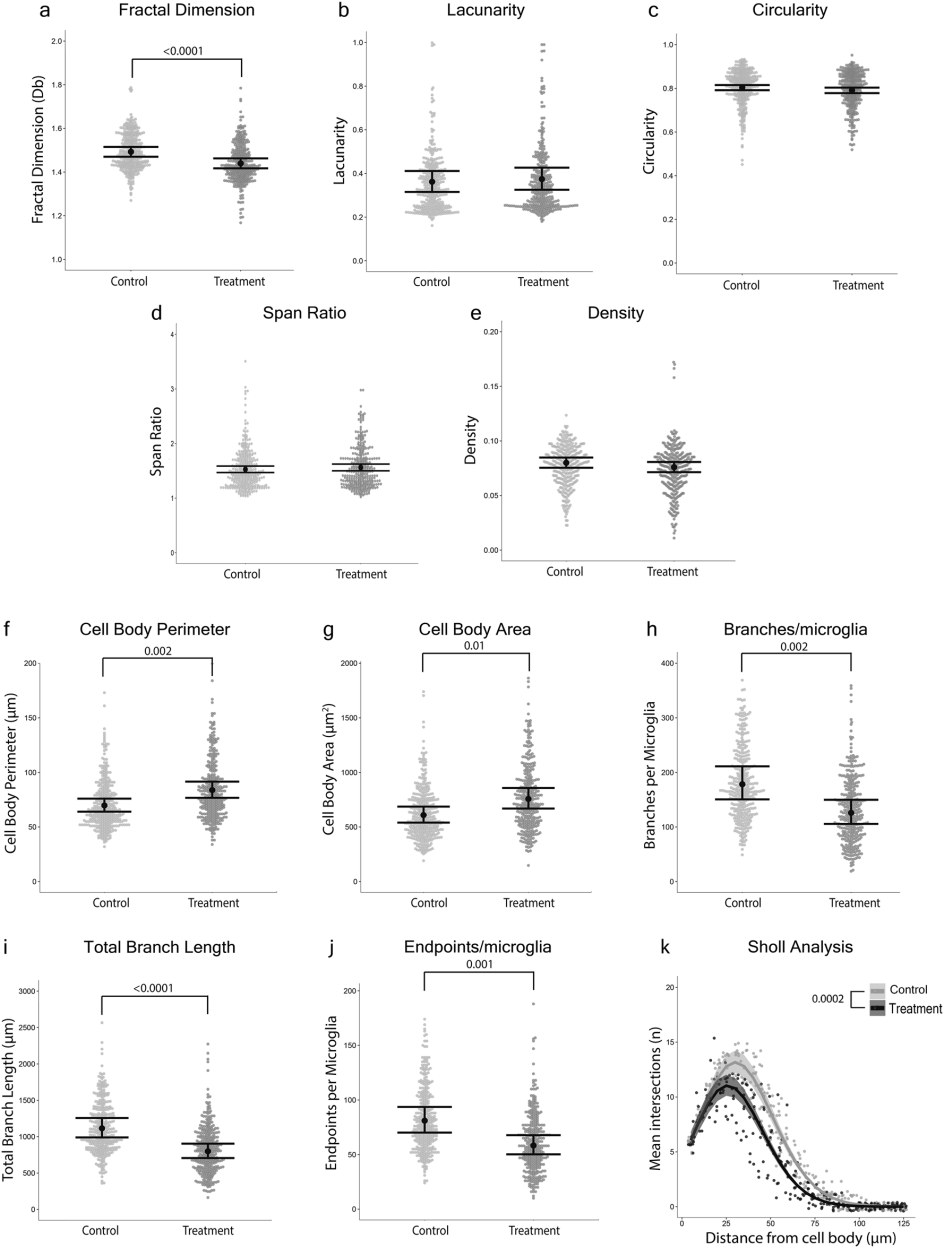
Quantitative methods that analyzed microglial morphology at a single cell level were sensitive to differences between control and treatment groups. Fractal analysis detected that microglia from the treatment group had a less complex branching pattern (fractal dimension) than controls. (**a**) Fractal dimension (Db) of microglia; (**b**) lacunarity of microglia; (**c**) circularity of microglia; (**d**) span ratio of microglia; and (**e**) pixel density per microglia. Individual data points are presented as gray dots. Results are presented as point estimates with 95% confidence intervals, which were estimated from mixed effects models with Beta, Gaussian (fractal dimension), or Gamma (span ratio) error distributions. Individual skeletal analysis detected that microglia from the treatment group had larger cell bodies and less ramification than controls. (**f**) Perimeter of cell bodies; (**g**) area of microglia cell bodies; (**h**) number of branches per microglial cell; (**i**) total branch length per microglial cell; and (**j**) number of branch endpoints per microglial cell. Individual data points are presented as gray dots. Results are presented as point estimates with 95% confidence intervals, which were estimated from mixed effects models with a negative-binomial error distribution. Sholl analysis detected that microglia from the treatment group were less ramified than controls. (**k**) The mean number of intersections on concentric circles every 5 µm from the cell body per microglial cell. Individual data points are presented as gray dots. Results are presented as point estimates with 95% confidence intervals, which were estimated from a mixed effects model with a negative-binomial error distribution. Treatment *n* = 324 microglia (12 mice, 27 microglia/mouse), Control *n* = 345 microglia (13 mice, 27 microglia/mouse).

Fractal analysis performed on isolated microglia detected that microglia from the treatment group had a less complex branching pattern (fractal dimension) than controls (*p*-value < 0.0001, Mean ∆ = 0.05, *d* = 0.65; Fig. 4a). However, differences in lacunarity (*p*-value = 0.72, Mean ∆ = 0.013, *d* = 0.004), circularity (*p*-value = 0.08, Mean ∆ = 0.013, *d* = 0.003), span ratio (*p*-value = 0.33, Mean ∆ = 0.03, *d* = 0.10), and density (*p*-value = 0.22, Mean ∆ = 0.004, *d* = 0.0001) between treatment groups were not detected (Fig. 4b–e).

### Single cell skeletal analysis detected that microglia from the treatment group had larger cell bodies, fewer and shorter branches, and fewer endpoints than controls

Microglia from the treatment group had a larger cell body perimeter (*p*-value = 0.002, Mean ∆ = 14 µm, *d* = 0.01; Fig. 4f) and area (*p*-value = 0.01, Mean ∆ = 148 µm^2^, *d* = 0.02; Fig. 4g) than controls. Microglia from the treatment group had fewer branches per cell (*p*-value = 0.002, Mean ∆ = 52, *d* = 0.04; Fig. 4h), shorter branch length (*p*-value < 0.0001, Mean ∆ = 315 µm, *d* = 0.03; Fig. 4i), and fewer endpoints per cell (*p*-value = 0.001, Mean ∆ = 23, *d* = 0.03; Fig. 4j) than controls.

### Sholl analysis detected that microglia from the treatment group had fewer and less expansive branches than controls

Microglia from the treatment group had fewer branches intersecting the Sholl analysis circles than controls (*p*-value = 0.002, Mean ∆ = 2.29 intersections, *d* = 0.003; Fig. 4k).

## Discussion

The diverse range of microglial morphology is often used to measure physiological states in the brain^16,17^; thus, it is critical that microglial morphology is accurately quantified. We compared commonly used methods for examining microglial morphology and found multiple inconsistencies among the approaches (Table 1).

**Table 1:** Summary of comparisons among contemporary ImageJ-based methods used for quantifying microglial morphology.

Method	Pros	Cons
Percent coverage	Quick to perform Can be used to quantify many stains	No morphological data Very sensitive to changes in stain quality, background staining, and changes to stain protocols Averaging across photomicrographs can introduce bias
Full photomicrograph skeletal analysis	Quick to perform Provides some morphological data	Affected by changes in stain quality and background staining Thresholding photomicrographs to correct for background staining trims microglial branches and alters morphological data Averaging across cells in a photomicrograph can introduce bias
Fractal analysis	Mathematical/geometric measures of cell complexity Can focus on specific cells closest to an area of interest, or other cell types	Time consuming to perform Interpretation requires knowledge in mathematics and geometry
Individual skeletal analysis & cell body area/perimeter	Accurately detects morphological changes Assesses hallmark features of microglial morphological reactivity (cell body size and ramification) Quantitative measure of cell span Can focus on specific cells closest to an area of interest, or other cell types Easy to interpret	Time consuming and labor intensive to perform
Sholl analysis	Detects changes in ramification Quantitative measure of cell span Can focus on specific cells closest to an area of interest, or other cell types	Assumes circularity and Euclidean distances Difficult to place first ring because microglial cell bodies are noncircular, which can result in missed primary processes, and secondary processes erroneously recorded as primary processes

**Figure 5:**
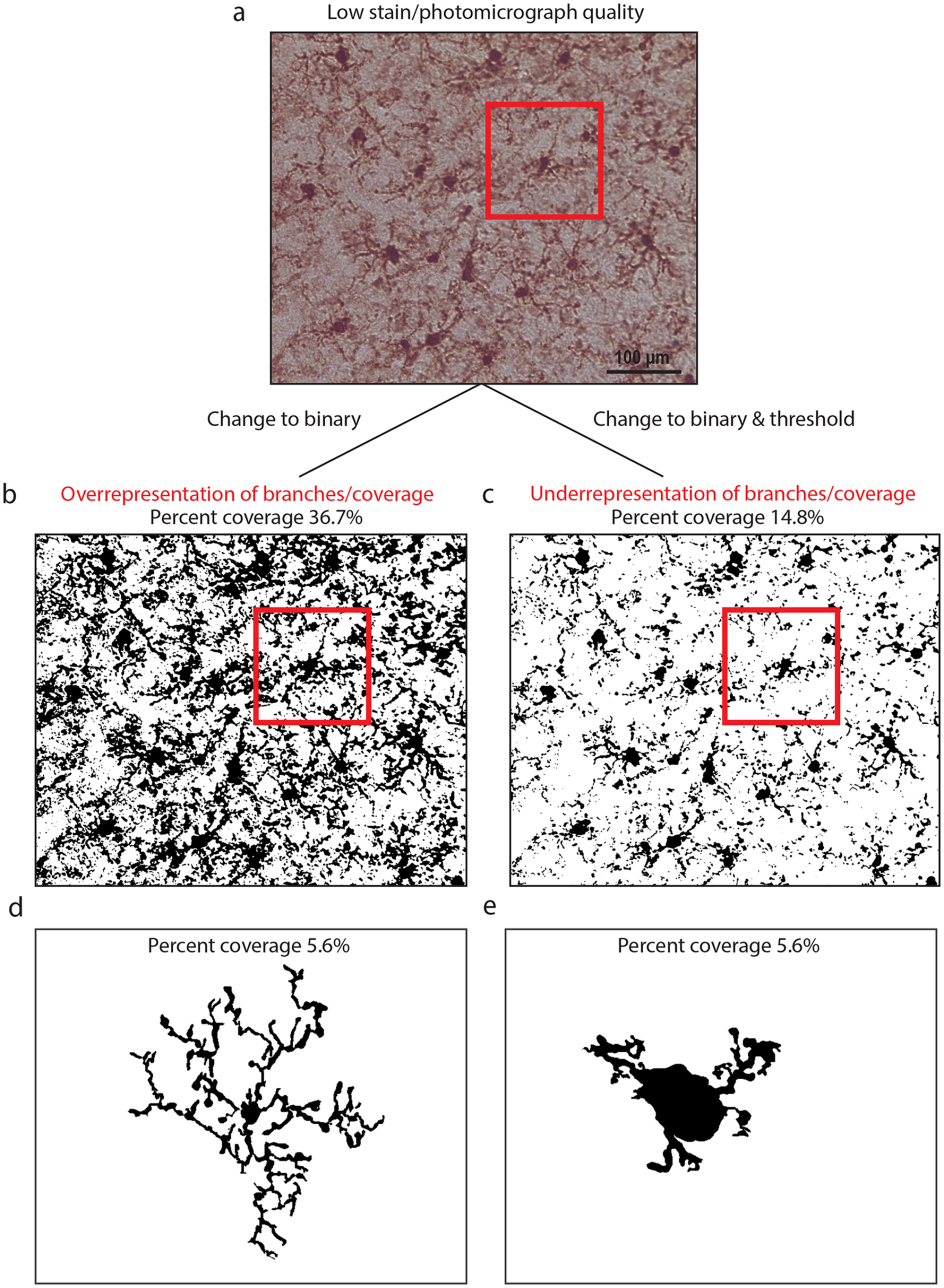
Photomicrograph manipulation can easily change percent coverage and full photomicrograph skeletal analysis results. (**a**) Example of a low-quality image. (**b**) Low-quality photomicrograph displayed in binary with no manipulation. Because of high background staining, microglia are overrepresented as a percent coverage of Iba1 staining. (**c**) When the threshold in B is adjusted to remove some background, some processes on microglial cells are lost and the cells appear less ramified. (**d**) A highly ramified microglial cell with 5.6% percent coverage. (**e**) A sparsely ramified microglial cell with 5.6% percent coverage.

Immunohistochemical data are easily compromised by mishandling and over processing of tissue samples and photomicrographs. Many immunohistochemical analyses rely on photomicrograph pre-processing. We demonstrated how changing photomicrograph parameters can change the output data and lead to inaccurate inferences (Fig. 5). For example, the percent coverage method is based on the principle that reactive microglia express higher levels of Iba1^18,19^. Figure 5a shows a low-quality photomicrograph (included for demonstrative purposes) that, when converted to binary (Fig. 5b), yields high percent coverage because of substantial background staining and image artifact. Consequently, photomicrographs with large amounts of background staining skew the data and result in overrepresentation of Iba1 expression. This overrepresentation is subsequently interpreted by researchers as a higher level of microglial reactivity which may not represent a true biological effect. If researchers attempt to account for the high background by manipulating the photomicrograph (i.e., threshold adjustments), microglial branches are removed, which yields lower percent coverage and fewer branches with shorter lengths per microglial cell (Fig. 5c). This skews the data in the opposite direction, and data are interpreted as having lower Iba1 expression, less ramification, and reactivity. In full photomicrograph skeletal analysis of branches, background manipulations can also make microglia appear less ramified (more reactive). Clearly, photomicrograph manipulations can change the data and result in erroneous conclusions that are not representative of true biological effects. Percent coverage data obtained from different staining methods and protocols, should not be compared within or among morphological studies. This has been shown in neurons, whereby different staining methods resulted in substantially different morphological measurements^20^.

Although full photomicrograph techniques are quick to perform, our findings suggest that this may be at the expense of critical morphological details. Although percent coverage techniques can show differences in the overall amount of staining, they do not provide any morphological information. For example, a highly ramified microglia with a small cell body covering 5.6% of the total image is shown in Fig. 5d. Yet, the cell shown in Fig. 5e also covers 5.6% of the total image despite having a larger cell body and shorter branches, representing a reactive morphology. Together, these images demonstrate that percent coverage alone does not provide information about microglial morphology/reactivity and instead simply reflects the amount of staining in a photomicrograph.

Our comparisons of results from the full photomicrograph skeletal analysis, single cell skeletal analysis, and the percent coverage models revealed pervasive “ecological fallacy” that could lead to erroneous inferences (i.e., the flawed assumption that what is true at the aggregate [population] level must be true at the lower [individual] level^21^). Specifically, we found substantial aggregation bias in the full photomicrograph skeletal analysis data and the mean percent coverage data that was caused by aggregating/averaging across microglia or photomicrographs from the same animal^22,23^. The issue was so severe in our example data that strong, statistically significant treatment effects in three of four single cell skeletal analysis metrics were masked in the equivalent full photomicrograph skeletal analysis metrics. Further, a statistically significant treatment effect on percent coverage was masked in the photomicrograph-averaged percent coverage (sensu Simpson’s paradox^24,25^). An additional problem with aggregating/averaging across microglia or photomicrographs to produce datasets is that the unit of inference can change, which researchers may be unaware of and consequently make specious conclusions. For example, averaging microglial endpoints in full photomicrograph skeletal analysis can result in the unit of inference being the field of view within which the microglia are contained rather than being the microglia where the effects occurred and were actually measured. Furthermore, our models revealed that considerable reductions of effect sizes may occur when averaging/aggregating is employed to produce morphological datasets, primarily because averaging/aggregating causes a substantial loss of information. For instance, the averaged metrics from full photomicrograph skeletal analysis (Fig. 3c,d,f) had 67–93% smaller effect sizes than the same metrics from the single cell skeletal analysis (Fig. 4h–j). The photomicrograph-averaged mean percent coverage (Fig. 3a) had a 70% smaller effect size than the photomicrograph-specific percent coverage (Fig. 3b). We therefore urge researchers to employ caution when considering using full photomicrograph skeletal analysis and mean percent coverage in microglial morphological studies. We also suggest that readers consider our findings when interpreting the results of previously published studies that assessed microglial morphology.

Both mean percent coverage and full photomicrograph skeletal analysis are quick to perform, making them appealing metrics for high throughput studies. However, both approaches are affected by staining quality, photomicrograph manipulations, and the consequences of averaging. Single cell microglial analysis techniques are more laborious, but provide detailed morphological data that, when analyzed using appropriate statistical methods that account for clustering of observations, allow for reliable inferences. Fractal analysis assesses cell complexity using mathematical and geometric methods of pattern complexity^26^. These detailed measures are useful to examine the branching patterns of microglia cells, which signifies reactivity status, but the resulting mathematical data require background knowledge to interpret how the results reflect microglial reactivity. Individual skeletal analysis, with cell body area and perimeter measures, gives a detailed account of the two hallmark morphological features of microglial reactivity (i.e., decreased branching and increased cell body size). Our results suggest that individual skeletal analysis can detect even small changes in microglial morphology, as all outcome measures showed that microglia from the treatment group had a more reactive morphology than controls. Another study that used cell body area and perimeter measurements to quantify microglial activation also found that these measures had a strong correlation with microglial activation^27^.

Sholl analysis was also able to detect changes in microglial morphology in the treatment group compared to control group; although, this was likely at least partially due to our analytical approach using multilevel models that included random intercepts and nonlinear effects, which represent a substantial improvement over conventional methods that are traditionally used for analyzing Sholl data. Nevertheless, Sholl analysis was originally developed for analyzing neurons, which typically have less variation in cell body shape and size than microglia; therefore, the first Sholl circle around the cell body of the microglia often touched a secondary branch because of the irregular cell body sizes, highly complex branching patterns, and considerable branch sinuosity of microglia. Inaccurate placement of the first circle can lead to small branches near the cell body going uncounted because those branches do not reach the first Sholl circle. Consequently, a tradeoff exists between prioritizing the placement of the circle to surround the entire cell body and measuring all branches accurately.

Other morphological quantification techniques exist that we did not include in this study. For example, nearest neighbor analyses are used to examine the spatial relationship between microglia^28^. 3D reconstruction of microglial cells in Neurolucida software produces data similar to single cell skeletal analysis that includes branch length, branch number, and cell body size^29^. Microglial reactivity can also be examined using genetic approaches such as RNA sequencing and NanoString gene expression analysis, which involve genetic profiling of isolated microglia or isolated brain regions^30,31^. These genetic approaches are critical in elucidating the function of microglia following an inflammatory stimulus.

In summary, percent coverage of Iba1 stain, one of the most common methods to quantify microglial morphology, was the least able to detect morphological differences between groups, particularly when the mean per animal was considered. This is, in part, due to the lack of morphological information that percent coverage provides. Skeletal analysis on full photomicrographs was plagued by aggregation bias that masked differences between treatment groups at the individual level. Importantly, skeletal analysis on isolated microglia provided the most comprehensive morphological data, which, when analyzed with hierarchical models, also produced the largest effect sizes for microglial morphology changes. Thus, we conclude that single cell analyses are more appropriate for detailed morphological studies, whereas full photomicrograph analyses might be useful for rapid screenings to investigate whether a treatment altered the microglial response, though we caution that aggregation bias could produce unreliable results. Our study should provide researchers with the necessary information to make study design and analytical decisions that result in more appropriate characterization of microglial morphology. We also note that the findings of our study might be applicable to other areas of neuroscience, because the morphology of neurons^15,32–34^ and astrocytes^12,35^ are commonly assessed using the same or similar immunohistochemistry techniques and metrics. Quantitative methods to describe microglial morphology should be selected based on several criteria including the research question, immunohistochemical experience of the researchers, and time required to isolate individual cells versus analyze full photomicrographs. Combinations of these techniques should be considered as they may give the most biologically accurate representation of microglial morphology.

## Methods

### Rigor

All animal studies were approved by the internal Institutional Animal Care and Use Committee (IACUC) at the University of Arizona (protocol 13-460) and were conducted in accordance with the National Institutes of Health (NIH) guidelines for the care and use of laboratory animals. Studies are reported following Animal Research: Reporting In Vivo Experiments (ARRIVE) guidelines. Animals were randomly assigned to manipulation groups before the initiation of the study to ensure equal distribution across groups. Data collection was performed by investigators that were blinded to the experimental conditions.

### Experimental design

Tissue samples analyzed in this study were generated from animals used in a previous study that examined the role of microglia in sleep after an immune challenge^36^. All data used in that study are publicly available in the Dryad digital repository^37^. This tissue was selected because the animal manipulations caused a robust difference in microglial ramification between treatment and control mice. Briefly, male C57BL/6J mice were randomly assigned to PLX (Plexxikon 5622 1200 ppm; formulated in AIN-76A rodent chow) diet to deplete microglia or control diet (AIN-76A rodent chow) for 21 days^38^. On day 21, mice were given an intraperitoneal lipopolysaccharide (LPS; *E. coli* 0111:B4, Sigma-Aldrich in sterile saline) injection at 0.4 mg/kg in a volume of 0.05 ml to induce an inflammatory challenge^39^. Four days post-LPS injection, all mice were returned to standard diet and were given 10 days for microglia to repopulate. After 10 days of repopulation, all mice were given a second LPS injection and maintained on the standard rodent chow. Brains were collected at 7 days following the second LPS administration.

### Perfusion and tissue processing

Seven days following the second LPS injection, a lethal dose of Euthasol® was administered. Mice were transcardially perfused with phosphate buffered saline (PBS). Brains were drop fixed in 4% paraformaldehyde for 24 h and then cryoprotected in successive concentrations of sucrose (15%, 30%). Using the Megabrain technique^40^, brains were frozen and cryosectioned at 40 µm in the coronal plane and were immediately mounted onto slides.

### Immunohistochemistry

Prior to staining, the slides were baked at 56 °C for 3 h. Slides underwent antigen retrieval (sodium citrate buffer PH 6.0 for 100 min). After washing with PBS, PAP pen was applied to slides. Slides were incubated in blocking solution (4% Normal horse serum [NHS], 0.1% Triton X-100 in PBS) for 60 min, followed by incubation in primary antibody solution (rabbit anti-Iba1; WAKO cat #019919741 at 1:1000 concentration in 1% NHS, 0.1% Triton X-100 in PBS) overnight at 4 °C. Slides were then washed in PBS and 0.1% tween 20, and were incubated in secondary antibody solution (biotinylated horse anti-rabbit IgG (H + L); vector BA-1100 at 1:250 concentration in 4% NHS and 0.4% Triton X-100 in PBS) 60 min at room temperature. Slides were washed in PBS and endogenous peroxidases were blocked by incubation in hydrogen peroxide for 30 min. After washing in PBS, ABC solution (Vectastain ABC kit PK-6100) was applied for 30 min, followed by a PBS wash. 3,3′-Diaminobenzidine [from Vector DAB peroxidase substrate kit SK-4100] was applied to the slides for 10 min. Slides were then placed in tap water and ethanol of increasing concentrations (70%, 90%,100%). After treating the tissue with Citrosolve, coverslips were applied using dibutyl phthalate polystyrene xylene mounting medium. Samples were stained in two batches and treatment and control samples were split evenly between staining batches to minimize variation in staining.

### Imaging

Z-stacked photomicrographs were taken using a 40× objective lens on a Zeiss Imager A2 microscope via AxioCam MRc5 digital camera and Neurolucida 360 software, with consistent microscope settings and Z-stack parameters. Three slices per animal were taken from between bregma and lambda and were imaged in the retrosplenial, somatosensory, and entorhinal cortices. A total of 669 microglia (randomly selected using coordinates and a random number generator) from 225 photomicrographs (345 microglia from 13 control mice, 324 microglia from 12 treatment mice) were analyzed. All analyses used the same photomicrographs and isolated microglia for direct comparison. Images with clearly visible microglial cell bodies were included in this study, to minimize staining/imaging artefact.

### Percent coverage photomicrograph analysis

The steps followed for percent coverage calculations were based on previous studies that used percent or pixel coverage techniques to indicate microglial reactivity^41–47^. Percent coverage analysis is often referred to as optical/pixel density or intensity of staining/fluorescence. Using Image-J, raw photomicrographs were converted to 8-bit and the ‘subtract background’ function was applied. To determine the rolling ball radius for the background subtraction, we took an average radius from 5 cell bodies and set the rolling ball radius at 50% of this radius. The same rolling ball radius of 50 pixels was used throughout the study. Then the photomicrograph (Fig. 1a) was converted to binary and minimal adjustments to the threshold were made so that the binary image best represented the raw data (Fig. 1b), and any processing artifact was filtered out. The percentage of the image covered by dark pixels was calculated. Data from each mouse included 9 cortical photomicrographs (3 brain slices per mouse, 3 photomicrographs per slice). We employed two approaches for generating percent coverage data: (1) The percent coverage values for the 9 photomicrographs were averaged to obtain a single percent coverage value per mouse, and (2) each of the 9 separate percent coverage values per mouse were retained as individual data points without averaging the values.

### Full photomicrograph skeletal analysis

Iba1 staining was analyzed using the skeletal analysis plugin following the protocol previously published^6,14,48^. In brief, photomicrographs were pre-processed by converting to 8-bit and applying the FFT bandpass filter in ImageJ. The brightness/contrast of the photomicrograph was then adjusted to best visualize the branches of the microglia. The unsharp mask was then applied to further increase the contrast of the photomicrograph, and the despeckle function was applied to remove pixels/noise. The threshold was then adjusted, and the despeckle, close, and the remove outliers functions were applied. The binarized image was then skeletonized (Fig. 1c). Microglial cell somas were counted manually to obtain a total microglial count per photomicrograph. The total microglial branch length, branch endpoints, and number of branches were calculated across the entire image (Fig. 1d–f) and then averaged by the number of microglial cells per frame.

### Fractal analysis

According to the previously published protocol, randomly selected microglia were isolated from the photomicrographs and underwent fractal analysis^6,14^. In brief, this involved converting the photomicrograph to binary (Fig. 2a,b), creating a region of interest (ROI) that was sized to fit around all microglia in the study to ensure the scale of the isolated microglia was the same. Using a random number generator, coordinates were used to randomly select 3 microglia per image (Fig. 2c). Using the ROI, each selected cell was removed from the binary image. The paintbrush tool was used to remove any fragments that were not attached to the cell and connect any branches that became fragmented due to image processing, using the original photomicrograph as a reference. Once microglia were isolated, the binary image was converted to an outline. The FracLac plug-in was used to analyze the cells, with ‘box counting’ applied and the ‘grid design Num G’ set to 4. The convex hull and bounding circle of the cell (Fig. 2d) were measured. The fractal dimension (a statistical measure of pattern complexity), lacunarity (a geometric measure of how a pattern fills space), circularity (how circular the microglial cell is), span ratio (longest length/longest width), and density (number of pixels/area) were measured.

### Single cell skeletal analysis

The same cells that were isolated for fractal analysis were skeletonized and analyzed with the skeletal analysis plugin in ImageJ (Fig. 2e). The number of branches, the total branch length, and the endpoints per microglia were calculated per isolated cell using the ‘analyze skeleton function’. The cell body area and perimeter were calculated using the multipoint area selection tool (Fig. 2f).

### Sholl analysis

Sholl analysis was performed in ImageJ on the same isolated cells that were used for fractal and skeletal analysis. A radius was drawn from the center of the cell body to the end of the longest branch to set the upper and lower limit for concentric circle placement. The first circle was set as close to the edge of the cell body as possible to ensure the cell body was not counted as an intercept on the circle. The distance between each circle was set at 5 µm for all cells. The number of times that the microglial branches intercepted each of the circles was calculated (Fig. 2g).

### Statistical analyses

To investigate microglial differences between the control and treatment groups, we fit hierarchical generalized linear mixed models^49,50^. Depending on the outcome variable, different error distributions were needed to accurately reflect the data scales and obtain reliable parameter estimates. Lacunarity, density, circularity, percentage coverage, and mean percentage coverage were all proportions or percentages bounded between 0 and 1, so we specified Beta error distributions^51^. Number of branches, branch lengths, number of endpoints, cell area, cell perimeter, mean number of branches, mean branch lengths, mean number of endpoints, and mean number of cells were all overdispersed integer counts, so we specified negative-binomial error distributions^52,53^. Fractal dimension was a continuous variable that was approximately normally distributed for which we specified a Gaussian error distribution. Although span ratio was also a continuous variable, it was severely left-skewed; therefore, we specified a Gamma error distribution with a log link function^12,49^. For the Sholl analysis, the mean number of intersections outcome was an overdispersed count variable for which we specified a negative-binomial error distribution.

In all models for all outcomes, we included a fixed effect for treatment. In the Sholl analysis, we also included a fixed effect for distance from cell body as well as a two-way interaction between treatment and distance from cell body. Based on the results of previous studies, we expected the effect of distance from cell body on mean number of intersections to be nonlinear. Although to our knowledge no prior studies have explicitly modeled this nonlinearity, we chose to do so to improve inference reliability. We did this by including a natural cubic spline on distance from cell body in the model^54^.

In all models for single cell outcome measures, we included random intercepts for individual animal crossed with random intercepts for brain region. This random effects structure appropriately accommodated the hierarchical clustering of multiple data points from each mouse and the hierarchical clustering of data points from each individual within the three brain regions (*n* = 9 data points within each of three regions from each individual [*n* = 27 total data points from each mouse])^49,50^. In the models for cell-aggregated widefield outcome measures, we included random intercepts for individual mouse crossed with random intercepts for the binned number of cells that were used to obtain the outcome value (*n* = 9 data points from each mouse)^36^. This random effects structure accommodated hierarchical clustering of multiple aggregate data points from each mouse and the possibility that the number of cells used to calculate a given value might introduce unaccounted for variation. In the Sholl analysis model, we included only a random intercept for individual mouse to accommodate the hierarchical clustering of multiple data points from each mouse (*n* = 25 distances at which the mean number of intersections was calculated from each mouse).

We fit all models in the frequentist framework using the package *glmmTMB* in the R statistical computing environment^55,56^. We based inferences on a combination of coefficient estimates (β) and their 95% confidence intervals, differences between predicted conditional means (∆), effect sizes (*d*)^57^, and *p*-values following Tukey’s adjustments for multiple comparisons^58^, all of which were obtained using the package *emmeans* in R^59^.

## Data Availability

All data supporting the conclusions of this article are publicly available in the Dryad Digital Repository at the following link: https://doi.org/10.5061/dryad.j3tx95xhq.
